# Nano-Emulsification of Cinnamon and Curcuma Essential Oils for the Quality Improvement of Minced Meat Beef

**DOI:** 10.3390/foods12020235

**Published:** 2023-01-04

**Authors:** Safa Dghais, Mariem Ben Jemaa, Maryem Chouchen, Selim Jallouli, Riadh Ksouri, Hanen Falleh

**Affiliations:** 1 Laboratory of Aromatic and Medicinal Plants, Biotechnology Center of Borj-Cédria, BP 901, Hammam-Lif 2050, Tunisia; 2 Laboratoire des Substances Bioactives, Biotechnology Center of Borj-Cédria, BP 901, Hammam-Lif 2050, Tunisia

**Keywords:** essential oils, nanoencapsulation, antibacterial activity, meat preservation

## Abstract

This work aims to evaluate cinnamon and curcuma essential oils as natural preservatives in minced beef meat. Essential oil chemical compositions and antibacterial activities were studied, and their encapsulation was optimized into nano-emulsions based on droplet size and distribution assessments. Selected formulas were further explored for their physical stabilities and antibacterial activities. Then, their effects on minced beef meat preservation were evaluated. Results showed significant differences in the chemical compositions and the efficiency of the tested essential oils, with cinnamon having a significant antibacterial efficacy. Formulation results showed that cinnamon nanoemulsion, encapsulated by 7.5% Tween 80, possessed an 89 nm droplet size, while the droplet diameter of curcuma nanoemulsion, encapsulated by 5% Tween 80, was 151 nm. Antimicrobial results depicted a significantly higher activity in nanoemulsions as compared to essential oils. For instance, the inhibition diameter of cinnamon essential oils against *S. aureus* was equal to 35 mm, while that of its nanoemulsion reached 40 mm. The meat preservation results showed that both bulk and nanoencapsulated essential oils significantly inhibited bacterial growth, as well as the formation of methemoglobin and lipid oxidation in meat. Thus, this work draws attention to the enhanced preservation effects of essential oils on the processing of minced beef meat as well as the great potential of nanoemulsions as carriers for essential oils in food industry applications.

## 1. Introduction

Food is unquestionably crucial for living. Food safety is consequently a vital issue for both consumers and food industry. Food matrices such as meat and meat products are an ideal environment for the growth of destructive food-borne pathogens, which may constitute a threat to public health [1]. In this respect, the correct preservation of meat is challenging for food producers, not only because meat’s composition makes it a perishable material, but also due to the fact that meat’s sensory properties are highly sensitive to common sterilization processes [2]. Indeed, the increasing requirements for fresh/safe products are a challenge for meat producers consisting of providing safe food with minimal processing. Considering that some meat products are eaten medium-cooked or even raw, it is crucial that this food should be free of microbial contamination. Additionally, in this epoch of instant information transmission, consumers are increasingly aware that they may be exposed to potential health issues and cancer-causing agents when eating thermally processed meat products [2]. Therefore, finding workable solutions which are safe for human health is essential.

Plants are a source of bioactive molecules and efficient natural antimicrobials such as essential oils (EOs) [3]. EOs are bioactive secondary metabolites extracted from aromatic plants, generally through hydrodistillation, without any solvent or artificial additives [4]. These bioactive molecules are commonly known for their significant biological activities, particularly antimicrobial, and they exhibit the advantage of being generally recognized as safe or GRAS [1,4]. For these reasons, EOs have recently gained scientific interest for their capacity to prevent and reduce the threat of microorganisms and food spoilage [3,5]. At this point, it is important to highlight that EOs are volatile chemicals, and they are hydrophobic, highly unstable, and very fragile, as they are easily damaged in the presence of light, air, or high temperatures [6]. Consequently, using EOs as food preservatives is neither economically nor practically ideal if they are not protected from these external factors. With this in mind, encapsulating EOs into nanoemulsions (NEs) is an efficient means to shield them from environmental conditions, reduce their toxicity, and mask their marked flavor and noticeable taste. Additionally, the encapsulation of these bioactive molecules facilitates their homogeneous incorporation into different food matrices by reducing their hydrophobicity [6]. The encapsulation of EOs further aims to optimize the distribution and stability of preservative molecules in food products [7]. NEs ameliorate EOs’ efficacy in food matrices where target pathogenic microorganisms are preferentially located. Their extremely reduced size (nano scale) increases their active droplet surface area, and therefore, NEs are assumed to have higher antimicrobial activity than conventional emulsions with significantly higher droplet sizes [4].

The objective of this work was the valorization of bulk and encapsulated EOs as natural preservatives in the food industry. It first proposes to identify the main components of cinnamon and curcuma EOs and to assess their antimicrobial activities; subsequently, their stabilization in nanospheres and the physical and microbiological characterization of the newly formed nano-emulsions will be examined. Finally, the third part of the work will be devoted to the study of the preservative effects of bulk and encapsulated cinnamon and curcuma EOs when incorporated in minced beef meat. 

## 2. Materials and Methods

### 2.1. Plant Treatment and EOs Extraction

Dried cinnamon bark and curcuma rhizomes were purchased from a local market and were cut into small pieces before extraction. Samples were subjected to hydrodistillation using a Clevenger. The resulting EOs were stored in a refrigerator at −5 °C for further analyses.

### 2.2. GS-MS Characterization

The GC-MS apparatus, which was used to analyze EO samples, consisted of a gas chromatograph (HP-7890 A) coupled to a mass spectrometer (HP-MSD 5975 C) equipped with an RTX-5MS 5% phenyl methyl silox polar column (30 m length; 0.25 mm internal diameter; 0.25 μm film thickness). Helium (1 mL/min) injection was set in the split made (1/10). The temperature of the Injector was 250 °C, while the detector was set at 280 °C. Spectral data were acquired in the scan mode in the *m/z* range of 50 to 550 [8]. 

### 2.3. EOs Encapsulation

**Formulation protocol**. Different combinations were tested for the encapsulation of *C. zeylanicum* and *C. longa* EOs into nanoemulsions. The continuous phase of the formulated nanoemulsion (NE) was prepared by dissolving an emulsifier (Tween 40 or Tween 80) in deionized water at different concentrations. The dispersed phase (*C. zeylanicum* or *C. longa* EOs) presented 8 or 10% of the final emulsion weight. Experiment conditions were listed in Table 1. The most suitable formulations, in terms of droplet size average and polydispersity index, were considered for Zeta potential measurements (using Zetasizer Software, version 7.03) and for further analysis [4]. 

**Antibacterial activity measurements**. The antimicrobial potencies of *C. zeylanicum* and *C. longa* EOs and NEs were assessed against 4 food-borne bacteria: *Pseudomonas aeruginosa* (ATCC 27853), *Escherichia coli* (ATCC 8739), *Staphylococcus aureus* (ATCC 25923), and *Enterococcus hirae* (ATCC 10541), using the disc diffusion method. The inocula of each microorganism were streaked onto Mueller–Hinton agar plates. Next, sterile filter discs were impregnated with 10 µL of *C. zeylanicum* or *C. longa* EOs samples and placed on the inoculated agar. After incubation (37 °C for 24 h), the diameter of the inhibition zones around each disc was measured [9]. The vehicle control (deionized water + Tween 80) was tested for interference avoidance, and there was no perceptible inhibition of bacterial growth with it. According to the obtained results, the EO more efficient in inhibiting the growth of the tested pathogenic bacteria will be subjected to further investigation concerning the effect of encapsulation on the antibacterial activity. 

### 2.4. Incorporation of EOs and NEs in Minced Beef Meat

#### 2.4.1. Samples Preparation

The preparation of samples was completed according to method of Smaoui, et al. [10] with slight modifications. Fresh, raw minced beef was purchased from a local supplier. At room temperature, meat samples were divided into five equal portions of 50 g each and were incorporated with the equivalent of 1.5 mg/mL of cinnamon and curcuma (NE volume equal to 937.5 µL or EO volume equal to 75 µL) and a negative control (meat sample without EO or NE incorporation). All meat samples were stored at room temperature in non-aseptic conditions for 24 h and then analyzed for quality parameters.

#### 2.4.2. Analysis of Meat Samples

**pH**. Five grammes of each meat sample were homogenized with 50 mL of pure water (1:10, *w/v*) then filtered, and pH was measured using a pH meter. 

**Color**. Samples’ color was measured using a Lovibond PFX-i Series Spectro Colorimeter.

**Thiobarbituric acid reactive substances (TBARS) value**. Meat samples (2 g) were mixed with 100 μL of butylatedhydroxytoluene (1 g/L) and trichloroacetic acid (50 g/L) and were then homogenized by blender for 10 min and subsequently filtered [10]. Next, 2 mL of the filtrate were added to 2 mL of thiobarbituric acid solution. The tubes were then heated at 70 °C for 30 min and swiftly cooled in ice. The absorbance measured at 532 nm was corrected for the baseline drift as follows:

532 nm corrected = (532 nm − [(508 nm − 600 nm) × (600 − 532)])/[(600/508) − 600 nm] × 100

Results were expressed as mg of malonaldehyde equivalents per kg of sample (mg/kg).

**Metmyoglobin (MetMb) analysis**. Five grammes of meat samples were homogenized in 25 mL ice-cold phosphate buffer (40 mM, pH 6.8) for 10 s [10]. The mixture was kept at 4 °C for 1 h and then centrifuged at 4500 g for 30 min at 4 °C. The supernatant was filtered, and the absorbance was measured at 572, 565, 545, and 525 nm by a spectrophotometer. The MetMb percentages were calculated as follows:

MetMb (%) = [−2.51 + A572/A525 + 0.777 A565/A525 + 0.8 A545/A525 + 1.098] × 100

### 2.5. Statistical Analysis

At least three replicates were used for all tested parameters. Means were compared using the Duncan test at the *p* < 0.05 level, when significant differences were found using the Statistical package SAS 9.1 (2002, 525). Pearson’s correlation test was conducted in order to determine the linear correlation among the variables. A value of *p* < 0.05 was considered significant.

## 3. Results and Discussion

### 3.1. EOs Characterization

**EO yields**. The obtained EOs of Cinnamomum zeylanicum and Curcuma longa were pale-yellow liquids with a strong characteristic odor. The yield of Curcuma was higher than that of cinnamon, 0.7% and 0.5%, respectively. The cinnamon bark yield matches that recorded earlier by [11], being between 0.4 to 1.7% EO. Concerning curcuma EO, the results obtained by Loc, et al. [12] are superior to those obtained in this study (1.5%). These differences in EOs yields may be due to several factors, such as the geographical origin, climatic factors, the plant species itself, the growth stage, etc. [1].

**EOs composition**. The results in Table 2 suggest that cinnamon EO is composed of 13 different compounds in different amounts. As expected, cinnamaldehyde was the major molecule, representing 77% of the total components, distantly followed by α-terpineol (3.45%) and trans-cinnamyl acetate (3.31%). This result confirms those found by Falleh, Ben Jemaa, Djebali, Abid, Saada, and Ksouri [11] and Aisyah, et al. [13], who reported that cinnamaldehyde is the main constituent of cinnamon EO and can even reach 94% of the EO. Regarding curcuma EO, Table 2 reveals that 19 components have been identified, representing 97% of the total EO. According to Table 2, ar-turmerone was the major compound in curcuma EO at 73.5%, distantly followed by the monoterpene phellandrene (4.1%). This result agrees with those mentioned by Sandeep, et al. [14] who studied nine different varieties of curcuma and found that despite the agro-climatic differences, turmerone was always the main constituent of curcuma EO, with percentages varying from 40% to 75%.

### 3.2. Nanoemulsions Formulation and Characterization

#### 3.2.1. Optimization of Cinnamon and Curcuma NEs

The obtained results (Table 3) showed that nanoemulsions presented droplet size averages ranging from 842.47 to 89 nm with satisfying PDI values (below 0.6). Considering cinnamon EO, the best nanoemulsion formula (d3,2 = 89 nm and PDI = 0.32) was obtained using 8% dispersed phase + 92% aqueous phase containing 7.5% Tween 80 homogenized for 1 min by ultra-turrax. Aisyah, Haryani, Safriani, and El Husna [13] used a high-pressure homogenizer with Tween 80 at 20% to produce cinnamon nanoemulsion. The droplet diameter obtained under those conditions was 117.5 nm and was accompanied by a PDI equal to 0.123. These differences are probably due to the different energy sources, emulsifier (type and concentrations), and dispersed phase composition used for each study. Considering curcuma EO, the analysis in Table 3 revealed that curcuma NE with the lowest particle diameter (215.23 nm) and the best PDI (0.247) is obtained when using 8% dispersed phase in 92% of 5% Tween 80, homogenized by the combination of two physical forces (30 min of sonication + 1 min ultra-homogenization). The obtained results are in good agreement with the literature, since low-mass surfactants such as Tween 80 are known to rapidly coat the surface of the created oil–water interface during emulsification and reduce interfacial tension, thereby preventing droplet coalescence [15]. At the same time, sonication (ultrasonic waves at a high frequency) and ultra-homogenization (intensive and instant grinding of liquid droplets) are usually recommended to obtain the smallest possible particle sizes [16].

**Figure 1:**
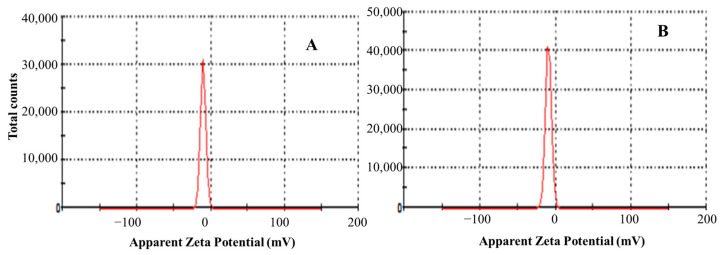
Zeta potential of curcuma (**A**) and cinnamon (**B**) nanoemulsions. Results are the means of at least three replications.

Zeta potential is one of the most important parameters when assessing NE stability. According to Sari, et al. [17], a zeta potential of ±30 mV is believed to be sufficient for ensuring nanoemulsion physical stability. In the present case, the obtained results are monomodal (Figure 1). The obtained zeta potentials were −10.2 and −9.26 mV (cinnamon and curcuma NEs, respectively), indicating stable and homogeneous formulations.

#### 3.2.2. EO and Nanoemulsion Antibacterial Activities

*C. zeylanicum* EO was very active against the tested microbial strains, generating an inhibition diameter (ID) over 33 mm. Table 4 demonstrates that the efficiency of cinnamon EO is significant and reaches 43 mm (against *Esherichia coli*), which is statistically (*p* < 0.05) way better than the positive control (Ampicillin, 15 mm). This superiority of cinnamon EO over Ampicillin was confirmed with the four tested bacteria. Conversely, curcuma EO activity was limited, and the largest ID was equal to 8.66 mm (against *Enterococcus hirae*). Many other studies also confirmed that cinnamon NE presented better antibacterial capacity then bulk EO.

Indeed, when encapsulated in the hydrophilic NE, cinnamon EO diffusion would be considered homogeneous and its antimicrobial activity more efficient. In this regard, EO antibacterial activity is influenced by its compounds’ chemical structure, their proportions, as well as by their combined actions at several bacterial levels [18]. Accordingly, the high antimicrobial activity of cinnamon EO is probably correlated with its richness in cinnamaldehyde, one of the most active aldehydes, able to inhibit the growth of bacterial enzymes and cause damage to their cell walls [11]. Possible mechanisms of EOs against bacterial cells reported in the literature are (1) degradation of the cell wall, (2) damage to the cytoplasmic membrane, (3) damage to membrane proteins, (4) leakage of cell contents, and (5) coagulation of the cytoplasm and depletion of the proton motive force [1].

#### 3.2.3. Preservative Effect of Cinnamon and Curcuma EOs and NEs in Minced Meat

**Figure 2:**
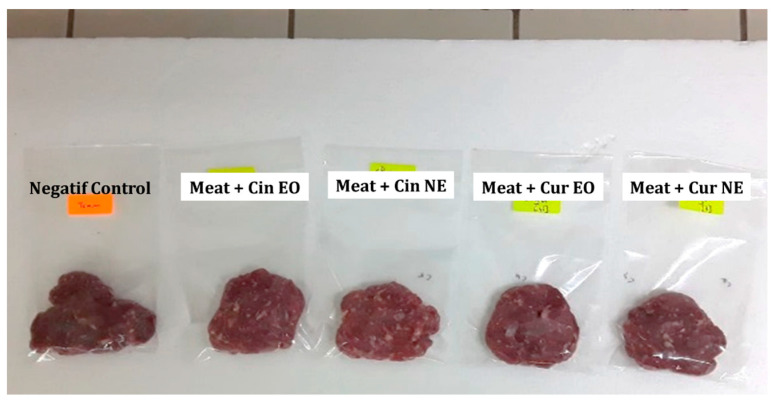
Visual aspects of minced meat (negative control) and minced meat treated with cinnamon essential oil (Cin EO), cinnamon nanoemulsion (Cin NE), curcuma essential oil (Cur EO) and curcuma nanoemulsion (Cur NE) after 24 h of storage.

Minced meat was chosen as the food matrix in order to test the efficacy of bulk and nanoencapsulated cinnamon and curcuma EOs in food preservation. As can be seen in Figure 2, both cinnamon and curcuma EOs and NEs succeeded in preserving the visual aspects of the meat, as the treated samples were visually clearer than the control.

**Figure 3:**
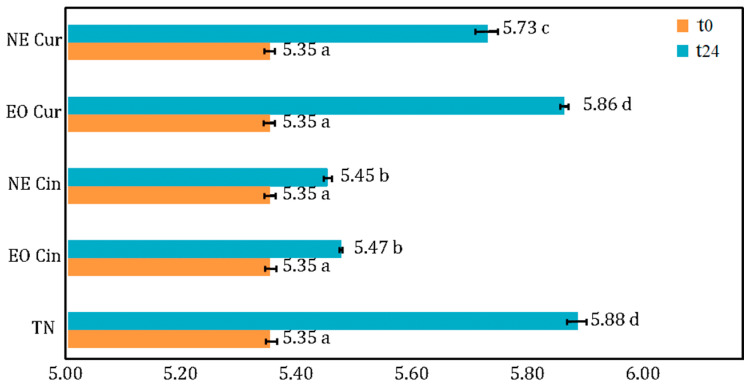
pH measurements of the control (TN) and minced meat treated with cinnamon EO (Cin EO), cinnamon NE (Cin NE), curcuma EO (Cur EO), and curcuma NE (Cur NE) at t = 0 and t = 24 h. Means with different letters were significantly different at the level of *p* < 0.05.

**pH measurement**. The pH values of the treated and untreated minced meat are shown in Figure 3. Treatment and time have a significant effect on pH evolution. In fact, the pH of the negative control rose from 5.35 (t = 0) to 5.88 at t = 24 h. Interestingly, curcuma EO exhibited the same pH at t = 24 h as the negative control. Meanwhile, after 24 h, cinnamon EO and NE and curcuma NE all managed to keep the pH of the meat statistically lower than control, between 5.45 and 5.73.

**Color measurement**. The surface color changes (L*, a*, b*) of the treated and control meat samples are shown in Table 5. For the negative control, the L* (lightness) values significantly increased throughout the storage period. The application of bulk and encapsulated cinnamon and curcuma oil effectively kept the L* values the same as the negative control at t = 0 h. Meanwhile, for the control meat, a* (redness) values significantly decreased between t = 0 h and t = 24 h (from 2.1 to 0.51, respectively), and significant differences were seen between the control and treated meat after 24 h. For the negative control, noticeable changes in the b* (yellowness) values were seen after 24 h, with the values decreasing from 22.92 to 33.48, while the b* values of the treatment group showed significant reduction during storage.

**Metmyoglobin assay**. Meat color strongly depended upon the chemical state of myoglobin. The analysis in Table 5 exhibited that the metmyoglobin percentage increased rapidly with 24 h of incubation and reached 76.24% in the control sample, whereas for treated samples, the MetMb percentage was braked and limited to 58.48% (curcuma EO). Interestingly, samples treated with cinnamon EO and NE presented no statistical difference (59.02 and 57.99%, respectively), while curcuma EO and NE showed no statistical differences (58.48 and 58.96%, respectively).

**TBARS assay**. Lipid oxidation is a primary factor limiting the shelf life of meat. This phenomenon can be estimated through the measurement of the MDA content in meat products. At t = 0 h, the TBARS values were found to be in the range of 0.081 mg of malonaldehyde/kg of sample. After 24 h, the TBARS values were significantly higher in the control (1.44 mg of malonaldehyde/kg) than in the treated samples. Moreover, cinnamon EO and NE and curcuma EO and NE TBARS values were kept low and comparable to day 0. Curcuma EO’s TBARS value was equal to 0.086, and similarly, its NE presents a malonaldehyde/kg value of 0.085 mg (Table 6).

### 3.3. Parameters Significance

The striated muscle is the main constituent of slaughtered animal carcasses [19]. After the animal’s death, the muscle undergoes many transformations which largely condition the final qualities of the meat. Meat is thus the result of the post-mortem evolution of skeletal muscle tissue. The evolution of the meat is carried out in three phases: the pantelance phase the cadaveric rigidity phase (during which rigor mortis occurs), and the maturation phase. The pantelance phase follows directly after slaughter. Its duration coincides with the duration of the survival of the nervous system and does not exceed 20 to 30 min [20]. Despite the interruption of blood flow, there is a succession of muscle contractions and relaxations. Indeed, the muscle continues to live. There is, therefore, an exhaustion of energy reserves followed by the establishment of anaerobic glycogenolysis. The ensuing accumulation of lactic acid thus causes the pH to drop [19] from 7 to 5.5. This fact explains the pH value that was obtained on the first day (control = 0 h), which was equal to 5.35. The maturation phase has classically been considered to constitute the phase of post-mortem evolution occurring after the onset of rigor mortis [20]. The disappearance of the energy reserves of the muscle and the acidification of the environment lead to protein denaturation [19]. Indeed, ambient temperatures (samples are stored at room temperature) associated with low pH (pH at t0 = 5.35), have been shown to cause an increase in the precipitation of sarcoplasmic proteins. Among these, myoglobin undergoes progressive denaturation during maturation, which results in a significant acceleration of its auto-oxidation [19].

It is important to note that pH is an important parameter influencing meat quality; the pH of beef muscle is around 7.0 at slaughtering and declines to 5.3 to 5.8 after 18 to 40 h [21]. In the present study, cinnamon and curcuma NEs managed to keep the pH of meat significantly lower than the control. This result reinforces that of Snoussi, et al. [22], who found that small beef meat pieces incorporated with thyme nanoemulsion produced the lowest pH values as compared to control sample without nanoemulsion (respectively 7 and 8.2 after 20 days of storage). Similarly, in another study, the pH of the control beef meat rose to an adverse level of 6.82, making steaks unfit for consumption, while pretreating steaks with 1% EOs of cumin, garlic, and thyme minimized pH fluctuations and preserved their viability for 18 days [23].

Myoglobin is the main pigment responsible for meat color [19]. Color is defined by three main parameters: hue (depending on the chemical state of the pigment), saturation (depending on the amount of pigment present in the muscle), and brightness (depending on the physicochemical characteristics of the muscle, which influences the diffusion of light). Meat color depended on the chemical state of myoglobin, a reduced form of which corresponds to the pigment of the deep muscle, the myoglobin (Fe^2+^) with red-purple color. On contact with air and cold, myoglobin combines with oxygen to form bright red oxymyoglobin, synonymous with the freshness of the meat [19]. Beyond a certain amount of time and influenced by the intrinsic properties of meat, such as temperature, light, and especially pH, the layer of oxymyoglobin disappears in favor of metmyoglobin, which is brown in color. The iron atom is then in its ferric (Fe^3+^) form [20]. According to our and previous results, EOs seem to be efficient in slowing down myoglobin oxidation to MetMb. Ben Akacha, et al. [24] monitored the evolution profile of MetMb in minced meat samples stored at 4 °C for 14 days and treated (or not) with essential oil. According to these authors, the development of MetMb in samples with the addition of Lobularia maritime EO was slower compared to BHT-treated meat, even for the lowest tested EO concentration, and the addition of L. maritime EO proved to extend the shelf life of stored meat by at least 7 days with respect to discoloration and protein oxidation [24].

As a whole, the increase in the pH values of all samples after 24 h is mainly due to the depletion of glycogen reserves [20]. This depletion induces an increase in the metmyoglobin levels, and therefore the meat turns brown, depending on the applied treatment. In the present study, the most effective treatment, therefore, is the one exhibiting the smallest increase in pH after 24 h. Accordingly, cinnamon EO and NE and curcuma NE are exhibit the best preservative power.

## 4. Conclusions

At the end of this work, it was found that the stable NEs formulated based on cinnamon and curcuma EOs improved their antioxidant and antimicrobial activities. Moreover, these formulated nano-emulsions exhibited pertinent preservation effects that may considerably extend the shelf life of ground meat. It can be concluded that bulk or nanoencapsulated cinnamon and curcuma EOs may be considered as promising tools for future application in meat products’ preservation.

## Figures and Tables

**Table 1:** Parameters of nanoemulsion formulation. Dispersed and aqueous phases percentages and compositions. EO: essential oil; emulsifier (T40: Tween 40; T80: Tween 80) and ultra-agitation and sonication conditions (min: minute).

	Dispersed Phase		Aqueous Phase		Extraction Conditions	
Exp N°	Percentage	Composition	Percentage	Composition	STIRRING (min)	SONICATION (min)
1	10	EO	90	1% T40	1	30
2	10	EO	90	5% T40	1	30
3	8	EO	92	5% T80	1	30
4	8	EO	92	7.5% T80	1	0

**Table 2:** Chemical composition of cinnamon and curcuma EOs (expressed as the % of total identified compounds) RT: retention time (in minutes).

N°	Chemical Formula	Compounds	RT (min)	% of Total Identified Compounds	
				Cinnamon EO	Curcuma EO
1	C_10_H_16_	α-pinene	7.872	1.74 ± 0.02	--
2	C_10_H_16_	Camphene	8.315	0.97 ± 0.01	--
3	C_7_H_6_O	Benzaldehyde	8.66	0.92 ± 0.1	--
4	C_10_H_16_	Phellandrene	10.042	--	4.10 ± 0.73
5	C_10_H_14_	p-Cymene	10.672	--	3.09 ± 1.05
6	C_10_H_16_	D-Limonene	10.797	0.95 ± 0.12	--
7	C_10_H_16_	Limonene	10.802	--	0.40 ± 0.01
8	C_10_H_18_O	1,8-Cineole	10.868	2.73 ± 1.06	2 88 ± 0.68
9	C_16_H_14_N_2_O_2_	BenzylAcetaldehyde	14.89	2.81 ± 0.52	--
10	C_10_H_18_O	Endo-Borneol	14.994	0.92 ± 0.02	--
11	C_10_H_18_O	α-Terpineol	15.73	4.48 ± 1.3	--
12	C_9_H_10_O	Cinnamyl alcohol	16.04	--	0.34 ± 0.01
13	C_9_H_8_O	Cinnamaldehyde	18.196	77.6 ± 4.65	--
14	C_12_H_18_	2,3-Dimethyl-2-phenylbutane	20.94	--	0.40 ± 0.02
15	C_15_H_24_	α-Copaene	20.893	0.60 ± 0.01	--
16	C_15_H_24_	Caryophyllene	22.042	0.44 ± 0.1	--
17	C_9_H_6_O_2_	Coumarin	22.422	0.91 ± 0.01	--
18	C_11_H_12_O_2_	Trans-Cinnamylacetate	22.597	3.31 ± 0.95	--
19	C_15_H_22_	Ar-curcumene	23.56	--	0.95 ± 0.01
20	C_15_H_24_	Zingiberene	23.87	--	0.53 ± 0.03
21	C_10_H_16_	β-Sesquiphellandrene	24.57	--	0.67 ± 0.07
22	C_8_H_9_N	2-Phenyl-1-Aziridine	25.88	--	2.03 ± 0.82
23	C_15_H_24_	trans-Caryophyllene	26.48	--	2.63 ± 0.1
24	C_10_H_16_O	β-Citral	26.54	--	0.46 ± 0.02
25	C_10_H_14_	o-Cymene	26.67	--	0.86 ± 0.01
26	C_10_H_16_	Camphene	26.95	--	0.31 ± 0.01
27	C_10_Cl_10_O	Curlone	27.15	--	0.39 ± 0.1
28	C_15_H_20_O	ar-Turmerone	27.21	--	73.5 ± 2.98
29	C_7_H_7_NO_2_	Benzaldehyde, 3-hydroxy-, oxime	28.70	--	0.8 ± 0.02
30	C_15_H_22_O	β-Atlantone	29.85	--	0.54 ± 0.01
31	C_15_H_22_O	α-Atlantone	30.09	--	2.17 ± 0.51
	Total (%)			98.38	97.02

**Table 3:** Cinnamon (Cin) and curcuma (Curc) nanoemulsion formulation characteristics: *d*_3,2_—average droplet size expressed in nm; PDI—polydispersity index.

Exp N°	Cin NE		Curc NE	
	*d*_3,2_	PDI	*d*_3,2_	PDI
1	322.57 ± 14.3 b	0.59 ± 0.01 c	2890 ± 34.13 d	0.84 ± 0.03 c
2	842.47 ± 10.3 c	0.38 ± 0.02 b	699.4 ± 37.43 c	0.41 ± 0.01 b
3	308.27 ± 11.7 b	0.23 ± 0.02 a	215.2 ± 32.61 a	0.25 ± 0.00 a
4	88.70 ± 9.43 a	0.32 ± 0.01 b	505.7 ± 32.87 b	0.42 ± 0.02 b

Results are the averages of at least three determinations. Different letters within a column indicate a significant difference (*p* > 0 05) between samples.

**Table 4:** The inhibition zone diameter, expressed in mm, of *C. zeylanicum* and *C. longa* essential oils (EO) and nanoemulsions (NE) as well as a positive control (ampicillin) against four bacterial strains.

Microbial Strains	Inhibition Zone (mm)				
	Cinnamon EO	Cinnamon NE	Curcuma EO	Curcuma NE	Ampicillin
*S. aureus*	34.67 ± 2.07 d	40.33 ± 4.09 e	6 ± 0.0 a	8.33 ± 0.88 b	16 ± 2.65 c
*E. hirae*	31.33 ± 2.86 c	35 ± 1.11 d	8.66 ± 1.03 a	12.67 ± 1.43 b	12.33 ± 2.6 b
*E. coli*	43 ± 3.67 d	48.67 ± 4.87 e	6.33 ± 0.03 a	9 ± 1.67 b	14.66 ± 1.7 c
*P. aeruginosa*	35.33 ± 1.98 d	36 ± 1.77 e	8 ± 0.97 a	10.33 ± 2.73 b	15.33 ± 3.22 c

Results are the means of at least six replications, and values followed by the same letter within a line are not significantly different at a 5% confidence level.

**Table 5:** Color and metmyoglobin (MetMb, expressed as %) assessments of the control (t = 0 and t = 24 h) and minced meat treated with cinnamon EO, cinnamon NE, curcuma EO, and curcuma NE for 24 h.

Meat Samples	MetMb (%)	Color		
		L*	a*	b*
Control 0 h	50.86 ± 9.12 a	65.77 ± 12.03 a	2.1 ± 0.08 f	22.92 ± 2.42 a,b
Control 24 h	76.24 ± 10.22 e	73.98 ± 9.77 d	0.51 ± 0.03 a	33.48 ± 1.44 c
Cin EO	59.02 ± 9.89 d	68.27 ± 8.34 c	1.50 ± 0.24 d	22.04 ± 2.05 a
Cin NE	57.99 ± 7.13 b	67.28 ± 7.54 b	1.71 ± 0.07 e	23.09 ± 2.99 b
Cur EO	58.48 ± 9.04 c	67.28 ± 8.89 b	1.21 ± 0.03 b	22.68 ± 1.56 a,b
Cur NE	58.96 ± 8.11 c,d	65.85 ± 7.12 a	1.39 ± 0.09 c	23.28 ± 2.75 b

Results are the means of at least three replications. Means with different letters within a column were significantly different at the level of *p* < 0 05.

**Table 6:** TBARS (expressed as mgMDAeq/kg) assays of the control (t = 0 and t = 24 h) and of the minced meat treated with cinnamon EO, cinnamon NE, curcuma EO, and curcuma NE for 24 h.

Meat Samples	Control		Cin		Cur	
	T = 0 h	T = 24 h	EO	NE	EO	NE
TBARS (mgMDAeq/kg)	0.087 ± 0.0 cd	0.092 ± 0.01 e	0.088 ± 0.01 d	0.082 ± 0.0 a	0.086 ± 0.0 bc	0.085 ± 0.0 b

Results are the means of at least three replications. Means with different letters were significantly different at the level of *p* < 0.5.

## Data Availability

Data is contained within the article.
